# The Predictive Value of Right Ventricular Longitudinal Strain in Pulmonary Hypertension, Heart Failure, and Valvular Diseases

**DOI:** 10.3389/fcvm.2021.698158

**Published:** 2021-06-17

**Authors:** Marijana Tadic, Nicoleta Nita, Leonhard Schneider, Johannes Kersten, Dominik Buckert, Birgid Gonska, Dominik Scharnbeck, Christine Reichart, Evgeny Belyavskiy, Cesare Cuspidi, Wolfang Rottbauer

**Affiliations:** ^1^Klinik für Innere Medizin II, Universitätsklinikum Ulm, Ulm, Germany; ^2^Department of Cardiology, Charité-University-Medicine (Campus Virchow – Klinikum), Berlin, Germany; ^3^Department of Medicine and Surgery, University of Milan-Bicocca, Milan, Italy

**Keywords:** right ventricle, longitudinal strain, outcome, pulmonary hypertension, heart failure, aortic stenosis, mitral regurgitation, tricuspid regurgitation

## Abstract

Right ventricular (RV) systolic function has an important role in the prediction of adverse outcomes, including mortality, in a wide range of cardiovascular (CV) conditions. Because of complex RV geometry and load dependency of the RV functional parameters, conventional echocardiographic parameters such as RV fractional area change (FAC) and tricuspid annular plane systolic excursion (TAPSE), have limited prognostic power in a large number of patients. RV longitudinal strain overcame the majority of these limitations, as it is angle-independent, less load-dependent, highly reproducible, and measure regional myocardial deformation. It has a high predictive value in patients with pulmonary hypertension, heart failure, congenital heart disease, ischemic heart disease, pulmonary embolism, cardiomyopathies, and valvular disease. It enables detection of subclinical RV damage even when conventional parameters of RV systolic function are in the normal range. Even though cardiac magnetic resonance-derived RV longitudinal strain showed excellent predictive value, echocardiography-derived RV strain remains the method of choice for evaluation of RV mechanics primarily due to high availability. Despite a constantly growing body of evidence that support RV longitudinal strain evaluation in the majority of CV patients, its assessment has not become the part of the routine echocardiographic examination in the majority of echocardiographic laboratories. The aim of this clinical review was to summarize the current data about the predictive value of RV longitudinal strain in patients with pulmonary hypertension, heart failure and valvular heart diseases.

## Introduction

The right ventricle (RV) has a complex anatomy and position in the thorax and therefore its assessment has long been limited and included a few conventional parameters that are easy to measure. Studies reported a significant prognostic effect of these RV parameters in a wide range of cardiovascular (CV) conditions such as pulmonary hypertension, congenital heart disease, pulmonary embolism, heart failure, cardiomyopathies, and valvular disease (1–6). Interestingly, RV parameters were reported as significant independent predictors of outcome in the general population and patients with arterial hypertension (7, 8). The development of imaging techniques provided detailed insight into RV structure, function and mechanics. Cardiac magnetic resonance and 3D echocardiography significantly improved our knowledge about RV structure and function, but speckle-tracking imaging enabled efficient and prompt echocardiographic assessment of RV mechanics (9).

RV longitudinal strain represents the cornerstone of RV mechanics evaluation with excellent reproducibility and high predictive value in patients with different aforementioned CV diseases (10–13). It has higher sensitivity and specificity in the detection of subclinical RV damage in patients with cardiomyopathies, cardiac amyloidosis and cancer (14–16). Although cardiac magnetic resonance provides more possibilities, that include the accurate evaluation of RV volumes and function, its relatively low availability and high level of competence that this kind of imaging demands, represent the main obstacles for its adoption in everyday clinical practice. Echocardiography-derived RV strain remains the method of choice for the evaluation of RV mechanics. Despite a constantly growing body of evidence that support RV longitudinal strain evaluation in the majority of CV patients, its assessment has not become part of the routine echocardiographic examination in majority of echocardiographic laboratories.

The aim of this clinical review was to summarize the current data about the predictive value of RV longitudinal strain in patients with pulmonary hypertension, heart failure, and valvular diseases in order to emphasize the importance of its evaluation in routine echocardiographic examination in these conditions.

## Right Ventricular Strain—Basic Characteristics

The retrosternal position of the RV and its unique crescent-shaped geometry with inflow and outflow tracts in almost the same plane is challenging for echocardiographic assessment (17). Several echocardiographic views should be used for appropriate evaluation of RV structure and function and not all of them are part of the basic echocardiographic examination that underwent the majority of CV patients. Therefore, practically all parameters that are recommended by the guidelines (RV diameters, TAPSE, FAC, s′, E/e′, sPAP) are assessed in a 4-chamber view (18), which is easy to access.

**Figure 1:**
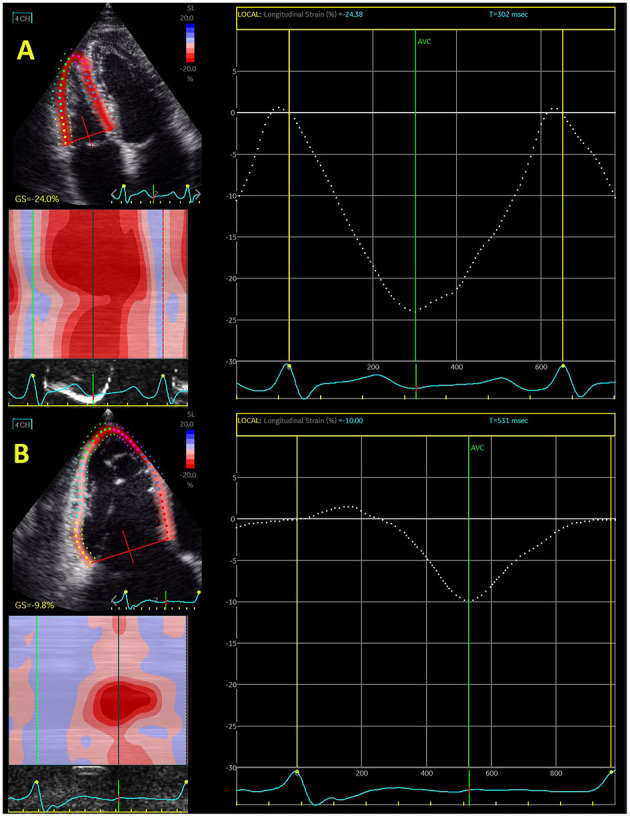
Echocardiography-derived right ventricular global longitudinal strain in control subjects **(A)** and patient with severe functional tricuspid regurgitation **(B)**.

The evaluation of RV longitudinal strain is also performed in 4-chamber view, which certainly represents an advantage because it does not demand additional time for acquisition (Figure 1). RV wall is significantly thinner than in the left ventricle (LV) and consists of only two layers that are predominantly longitudinally and obliquely directed in the free wall (17). The deep subendocardial fibers have longitudinal direction from base to apex, while the superficial subepicardial fibers normally have circumferential direction, parallel to the AV groove (19), but they turn obliquely as they approach to the apex of the heart and continue onto the LV. The continuity of RV and LV fibers significantly contributes to ventricular interdependence (19), which is important for both RV and LV function.

The technical aspects of image acquisition for speckle-tracking assessment are specific because they need to provide optimal images for post processing and high level of reproducibility for strain assessment. The suggested frame rate is between 40 and 90 frames per second for analysis of cardiac deformation. With increase of heart rate requires a higher frame rate to allow optimal speckle-tracking echocardiography. The acquisition of high-quality images is necessary for strain analysis, which considers the optimal gain settings and breath-hold techniques to clearly define the endocardial and epicardial borders and to avoid artifact associated to rib or lung motions and translational heart movement. RV focused apical 4-chamber view is necessary for RVLS evaluation. The optimal RV focused view should provide the visualization of the whole RV, the RV-free wall, and avoid foreshortening of the RV apex.

There is still no consensus about the region of interest that should be included in evaluation of RVLS. Some authors suggest a full RV chamber six-segmental model including the interventricular septum into the calculation of RVLS, whereas more authors accepted a three-segmental model, which involves only the RV free wall (20). The six-segment model is more feasible than three-segment model due to easier tracking of the whole RV. Absolute LS values derived from the free wall of the RV are significantly higher than from the whole RV. The authors that promote three-segmental model claim that the septum is traditionally seen as part of the LV and therefore should not be included into RVLS assessment.

The normal values of RVLS are still a matter of debate. Study that included significant number of patients reported lower RVLS values in men than in women, independently of the three- or six-segment model (21). Reference limits of normality for RVLS were −20% for men and −20.3% for women, whereas for RV free-wall LS limits were −22.5% for men and −23.3% for women (21).

There are several commercially available software platforms that enable post-process evaluation of raw data. EchoPac (GE) and TomTec are the most frequently used in clinical and research circumstances. EchoPac provided six-segment model and curve for each RV segment (including interventricular septum) and three-segmental model can be derived from these curves involving only segments of RV free wall. However, it is vendor dependent tool and can be used only for evaluation of echocardiographic images obtained by GE machines. TomTec is a vendor-independent software that can measure all echocardiographic images and provides automatically three-segment model for RV free-wall LS evaluation (separate values for free-wall RV and interventricular septum).

RV strain has several advantages: angle-independency, less load-dependency, and accuracy in measurement of regional myocardial deformation. The major limitation of 2D RV strain is motion-dependent speckles loss outside the imaging plane, especially due to excessive motion of the RV lateral wall. Nevertheless, this has been largely overcome with the introduction of software that provides dedicated RV longitudinal strain analysis and separate evaluation of RV free-wall longitudinal strain and RV global longitudinal strain. The important limitations are also lack of agreement about method that should be used for RVLS evaluation (three- vs. six-model) and unknown normal values for RVLS. Advantages and limitations are presented in Table 1. Even though all parameters of RV systolic function—TAPSE, FAC, and RV free-wall longitudinal function were reported as independent predictors of overall and CV mortality in patients with different CVD, RV free-wall longitudinal strain was proven to be the only independent predictor in analysis adjusted for multiple demographic, clinical and echocardiographic characteristics (22, 23). Study that used echocardiographic and CMR evaluation of RV function showed good correlation between TAPSE, s′, FAC, and global RV longitudinal strain and CMR-derived RV ejection fraction (24). Free-wall RV longitudinal strain showed the highest diagnostic accuracy and very high sensitivity and specificity to predict reduced EV ejection fraction (RVEF) <45% (24).

**Table 1:** Advantages and limitations for evaluation of RV longitudinal strain.

**Advantages**	**Disadvantages**
Additional parameter of RV systolic function	No agreement about normal values
High predictive value	No agreement about three- and six-segment models
High reproducibility	High temporal resolution necessary
Evaluation of mechanical function of the full RV wall thickness	Problem with imaging window and visualization of the RV free wall
Relatively load independent	Visualization of RV endocardial border sometimes can be challenging
Angle independent	Stable cardiac rhythm
High availability	
Low costs	
Short scan duration	
No need for advanced training	

*RV, right ventricle*.

## Rv Strain in Pulmonary Arterial Hypertension

The majority of studies investigated the predictive role of RV longitudinal strain in patients with idiopathic pulmonary arterial hypertension (PAH), but the number of researches that examined patients with pulmonary hypertension in pulmonary diseases (COPD, interstitial fibrosis) and connective tissue diseases (erythematous lupus, systemic sclerosis) is constantly increasing.

In patients with PAH, conventional parameters of RV systolic function, including TAPSE, FAC, s′, and RVEF, may be normal despite abnormal RV strain, which is an additional reason for the usage of RV strain in these patients. RV longitudinal strain can identify subclinical RV dysfunction at the early phase of disease and may serve as an important marker of subtle RV systolic dysfunction when conventional parameters, primarily TAPSE, are in the normal range (25, 26).

Small study that included 42 patients with PAH investigated the influence of different echocardiographic parameters on 4-year outcome and reported that RV free-wall longitudinal strain showed the best specificity and sensitivity in prediction of CV events in this population, significantly better than TAPSE, FAC, s′, and RV index of myocardial performance (27). PAH patients with RV free-wall longitudinal strain <-19.4% experienced fewer cardiovascular events than those with RV free-wall strain ≥-19.4% (27). Another study that investigated 51 PAH patients demonstrated that RV global longitudinal strain ≥-15.5% was independent predictor of adverse clinical events and mortality (28). The large study that included 575 patients with confirmed or suspected PAH reported significant decline in RV free-wall longitudinal strain proportional to worse functional class, shorter 6-min walk distance, higher N-terminal pro-B-type natriuretic peptide level, and the presence of right heart failure (29). RV free-wall longitudinal strain predicted survival after adjustment for pulmonary pressure, pulmonary vascular resistance, and right atrial pressure (29). Haeck et al. (30) involved 150 PH patients and demonstrated that RV free-wall strain ≥-19%, unlike TAPSE and FAC, was an independent predictor of all-cause mortality in these patients. All findings are summarized in Table 2.

**Table 2:** Predictive value of RV longitudinal strain in pulmonary hypertension.

**References**	**Sample size**	**RV free-wall/global GLS cut-off**	**Follow-up period (months)**	**Main findings**
Motoji et al. (27)	42	−19.4%	48	RV free-wall longitudinal strain showed the best specificity and sensitivity in prediction of CV events in this population, significantly better than TAPSE, FAC, s′ and RV index of myocardial performance.
Park et al. (28)	51	−15.5%	45 ± 15	RV global longitudinal strain ≥-15.5% was independent predictor of adverse clinical events and mortality.
Fine et al. (29)	575	−15%	18	RV free-wall longitudinal strain predicted survival after adjustment for pulmonary pressure, pulmonary vascular resistance, and right atrial pressure.
Haeck et al. (30)	150	−19%	31	RV free-wall longitudinal strain, unlike TAPSE and FAC, was independent an predictor of all-cause mortality in these patients.
Badagliacca et al. (31)	108	–	24	The authors reported that the shape of the RV strain curve and the longer time necessary to return RV strain to the baseline point were associated with worse outcome.
Hardegree et al. (32)	50	−12.5%	48	≥5% improvement in RV free-wall longitudinal strain had >7-fold lower mortality during the follow-up of 4 years.
Hulshof et al. (33)	1,169	−19%	–	RV free-wall strain >-19% had a significantly higher risk for the combined endpoint (mortality and PAH-related events).
Leng et al. (34)	80	−25.2%	24	CMR-derived RV longitudinal strain, TAPSE, FAC, and RA longitudinal strain were predictors of adverse outcome in these patients.
Sato et al. (35)	21	–	9	Improvement of CMR-derived RV strain during ambrisentan and tadalafil combination therapy in PAH patients with systemic sclerosis.

*CMR, cardiac magnetic resonance; CV, cardiovascular; FAC, fractional area change; GLS, global longitudinal strain; PAH, pulmonary arterial hypertension; RA, right atrium; RV, right ventricle; s′, systolic velocity of the lateral segment of tricuspid annulus; TAPSE, tricuspid annular plane systolic excursion*.

Badagliacca et al. (31) recently identified 3 different RV free-wall strain patterns in PAH patients based on the time period from peak-systolic strain to return to the baseline point-set for the basal and mid-RV free wall segments. The authors reported that the shape of the RV strain curve and the longer time necessary to return RV strain to the baseline point were associated with worse outcome. Therefore, pattern 3, which corresponded to slow and steady gradual movement of a strain-derived curve, had the worst event-free survival during follow-up of 24 months (31) (Table 2).

Not only RV strain at baseline, but also its change during therapy and follow-up is important for prognosis of PAH patients. Hardegree et al. (32) reported that ≥5% improvement in RV free-wall longitudinal strain had >7-fold lower mortality during the follow-up of 4 years.

Meta-analysis that involved 1,169 PH patients revealed that RV free-wall strain >-19% had a significantly higher risk for the combined endpoint (mortality and PH-related events), while patients with >-22% had a significantly higher risk for all-cause mortality (33). It showed that a relative reduction of RV longitudinal strain <10% was insignificant for survival after multivariate analysis, while a relative reduction >10% of RV longitudinal strain represented significant and independent risk factor of adverse outcome in PH patients (33) (**Table 4**).

The role of CMR-derived RV longitudinal strain in prediction of adverse outcome in PAH patients is insufficiently known because of scarce data. Recent study that included 80 PAH patients provided CMR analysis of all RV and right atrial (RA) structural, functional and strain parameters and showed that RV longitudinal strain, TAPSE, FAC, and RA longitudinal strain were predictors of adverse outcome in these patients (34). Sato et al. (35) showed the improvement of CMR-derived RV strain during ambrisentan and tadalafil combination therapy in PAH patients with systemic sclerosis (Table 3).

**Table 3:** Predictive value of RV longitudinal strain in heart failure.

**References**	**Sample size**	**RV free-wall/global GLS cut-off**	**Follow-up period (months)**	**Main findings**
Cameli et al. (45)	590	−15%	18 ± 11	Free-wall RV longitudinal strain was the strongest predictor of combined outcome, even stronger than LV global longitudinal strain, which support importance of RV strain in prognostic stratification in HF patients.
Hamada-Harimura et al. (46)	618	−13.1%	14	In patients with acute HF decompensation RV free-wall longitudinal strain was an independent predictor of cardiac events (CV death or unplanned hospitalization due to HF worsening).
Vizzardi et al. (47)	60	−18%	32 ± 13	RV free-wall longitudinal strain, but not TAPSE, FAC, and s′, was independent predictor of CV events (hospitalization and CV mortality) in HFrEF patients.
Carluccio et al. (48)	200	−15.3%	28	RV free-wall longitudinal strain was a better predictor than TAPSE in HFrEF patients with the best discriminatory value of RV free-wall longitudinal strain.
Kusunose et al. (49)	58	−16%	5	RV longitudinal strain in HFrEF patients was a good predictor of functional capacity improvement (VO2 peak) in HFrEF patients who were referred for cardiac rehabilitation.
Motoki et al. (11)	171	−14.8%	60	RV free-wall strain was a predictor of adverse CV events (death, cardiac transplantation, and hospitalization due to HF) independently of age, LVEF, tricuspid s′, E/e′ septal, and right atrial volume index in a population of patients with chronic HFrEF.
Houard et al. (50)	266	−19%	56	Echocardiography-derived RV free-wall longitudinal strain was a better predictor of overall and CV mortality than TAPSE, FAC, and CMR-derived RVEF, RV longitudinal strain.
Carluccio et al. (51)	288	−15.3%	30 ± 23	Global RV longitudinal strain did not remain independent predictor of composite outcome (all-cause death/HF-related hospitalization) in the models that included LV parameters and other RV parameters, whereas RV free-wall strain remained an independent predictor in all models.
Lisi et al. (52)	27	–	–	RV free-wall strain had the highest diagnostic accuracy for detecting severe myocardial fibrosis, much better than TAPSE, right atrial longitudinal strain and VO2 peak.
Park et al. (53)	799	−12%	32	In patients with acute HF was found that global RV longitudinal strain was a predictor of all-cause mortality regardless of LV global longitudinal strain and clinical characteristics.
Bosch et al. (54)	657	−15.3%	24	The authors reported that RV free-wall longitudinal strain, sPAP and their ratio were independent predictors of composite endpoint (mortality and HF hospitalization) in the whole population of HF patients.

*CMR, cardiac magnetic resonance; CV, cardiovascular; FAC, fractional area change; GLS, global longitudinal strain; HF, heart failure; HFpEF, heart failure with preserved ejection fraction; HFrEF, heart failure with reduced ejection fraction; LV, left ventricle; RV, right ventricle; s′, systolic velocity of the lateral segment of tricuspid annulus; sPAP, systolic pulmonary pressure; TAPSE, tricuspid annular plane systolic excursion*.

## RV Strain in Heart Failure

RV dysfunction is known indicator of poor survival in patients with heart failure (HF) (55). Conventional echocardiographic parameters for assessment of RV systolic function such as TAPSE and FAC have already been proven as independent predictors of adverse outcome in HF (56). Dini et al. (57) showed not only that a reduced RV function at baseline was independent predictor of poor outcome, but also that the reversibility of abnormal RV function, assessed by TAPSE, was related with a better prognosis, independently of LVEF improvement, LV reverse remodeling, and cardiac resynchronization therapy. Study that included patients after acute myocardial infarction reported that FAC, used for evaluation of RV systolic function, was associated with an increased risk of all-cause mortality, CV death, sudden death, HF, and stroke (58). Another investigation that used tissue Doppler parameter for evaluation of RV systolic function (s′) in patients with HF with preserved LVEF showed that s′ <5 cm/s was predictor of CV mortality, recurrent HF, and ischemic stroke (59).

Investigations proved that RV longitudinal strain is deteriorated at early stage of HF (3). Data from the literature indicate that RV longitudinal strain provides a more accurate and less preload dependent estimation of the overall performance of the RV in HF patients (3). The majority of data are focused on patients with HF with reduced LVEF (HFrEF) (45, 46, 60). However, data regarding HF with preserved LVEF (HFpEF) also emphasize the important role of RV longitudinal strain in these patients (3). Table 3 summarizes all findings in this field.

**Figure 2:**
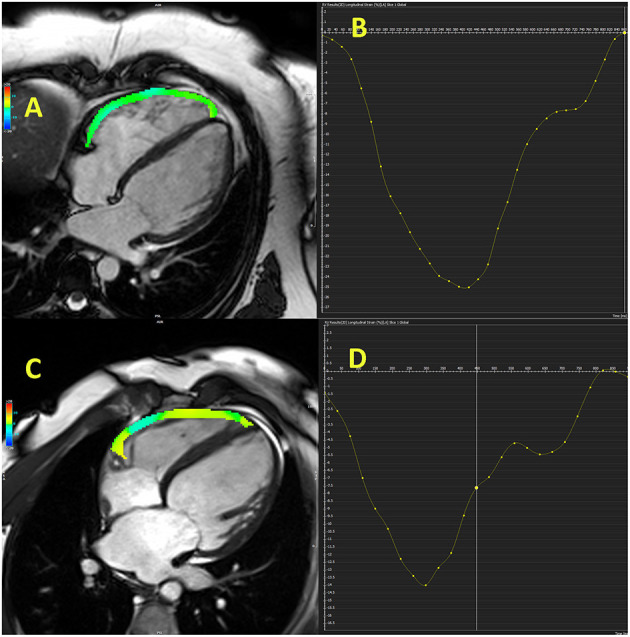
Cardiac magnetic resonance-derived right ventricular free-wall longitudinal strain in control subject **(A,B)** and patient with heart failure with reduced ejection fraction **(C,D)**.

Study that involved patients with HFrEF referred for heart transplantation reported that free-wall and global RV longitudinal strains, NT-pro-BNP, FAC, and LV end-diastolic volume were independent predictors of combined outcome (hospitalization for acute heart failure, cardiovascular death, heart transplantation, intra-aortic balloon pump implantation, and ventricular assist device implantation) (45). Free-wall RV longitudinal strain was the strongest predictor of combined outcome, even stronger than LV global longitudinal strain, which support importance of RV strain in prognostic stratification in HF patients (45). Another investigation showed that only RV free-wall longitudinal strain, but not TAPSE, FAC, and s′, was independent predictor of CV events (hospitalization and CV mortality) in HFrEF patients during 3-year follow-up (47). Carluccio et al. (48) revealed that RV free-wall longitudinal strain was a better predictor than TAPSE in HFrEF patients with the best discriminatory value of RV free-wall longitudinal strain for the prediction of adverse outcomes was −15.3%. Small study revealed that RV longitudinal strain <-16% in HFrEF patients was a good predictor of functional capacity improvement (VO2 peak) in HFrEF patients who were referred for cardiac rehabilitation (49). Another investigation found that RV free-wall strain was a predictor of adverse CV events (death, cardiac transplantation, and hospitalization due to HF) independently of age, LVEF, tricuspid s′, E/e′ septal, and right atrial volume index in a population of patients with chronic HFrEF who were followed for 5 years (11). The cut-off value used for RV free-wall strain was −14.8%. Study that combined CMR and echocardiographic evaluation of the RV in HFrEF patients showed that echocardiography-derived RV free-wall longitudinal strain was a better predictor of overall and CV mortality than TAPSE, FAC, and CMR-derived RVEF, RV longitudinal strain during 4.7-year follow-up period (50). Figure 2 illustrates RV free-wall longitudinal strain in control subjects and HFrEF patient.

Iacoviello et al. (60) demonstrated that both global and free-wall RV longitudinal strain were independent predictors of all-cause and CV mortality in chronic HFrEF patients during 3-year follow-up. Even though TAPSE, FAC, and s′ were predictors of mortality in univariate analysis, they were not included in multivariate analysis. On the other hand, Carluccio et al. (11) found superior predictive value of RV free-wall strain over RV global strain in HFrEF patients over 30-month follow-up. Global RV longitudinal strain did not remain independent predictor of composite outcome (all-cause death/HF-related hospitalization) in the models that included LV parameters (LVEF, LV longitudinal strain, left atrial volume index) and other RV parameters (tricuspid regurgitation severity, RV dimensions, systolic pulmonary pressure), whereas RV free-wall strain remained an independent predictor in all models (51) (Table 3).

The investigation that combined echocardiographic analysis with myocardial histologic analysis reported that RV free-wall longitudinal strain was the main determinant of myocardial fibrosis, which may explain high prognostic value of this parameter in HF patients (52). RV free-wall strain had the highest diagnostic accuracy for detecting severe myocardial fibrosis, much better than TAPSE, right atrial longitudinal strain and VO2 peak (52).

In patients with acute HF decompensation RV free-wall longitudinal strain was an independent predictor of cardiac events (CV death or unplanned hospitalization due to HF worsening) (46). Other echocardiographic parameters such as LVEF, TAPSE, FAC, RV and LV global longitudinal strains were not proven to be the independent predictors of outcome in these patients (46). The cut-off value for RV free-wall longitudinal strain that showed the highest sensitivity and specificity was −13.1% (46). In the similar group of patients with acute HF authors found that global RV longitudinal strain was a predictor of all-cause mortality regardless of LV global longitudinal strain and clinical characteristics (53). Patients with reduced biventricular longitudinal strain (>-9% for LV and >-12% for RV) had the worst prognosis (53). The predictive power of RV global longitudinal strain was more significant in the absence of PH (Table 3).

Bosch et al. (54) reported gradual and significant deterioration of RV longitudinal strain from controls across HFpEF to HFrEF patients. The authors reported that RV free-wall longitudinal strain, sPAP and their ratio were independent predictors of composite endpoint (mortality and HF hospitalization) in the whole population of HF patients (61). Unfortunately, the separate analyses of HFrEF and HFpEF patients were not performed and therefore predictive value of RV longitudinal strain in HFpEF remains to be determined.

## RV Strain in Valvular Heart DiseaseS

The effect of RV function in patients with valvular disease has been investigated in the last several years and it has become evident that effect of RV dysfunction on outcome in these patients is not irrelevant. The majority of studies reported deterioration of RV longitudinal strain in patients with valvular diseases (61, 62), but some recent investigations revealed a significant predictive value of RV strain in patients with aortic stenosis, mitral and tricuspid regurgitation (12, 13, 40, 63). Data are still scarce but warrant a special attention due to constantly increasing number of transcatheter interventions in valvular diseases and importance to develop new scores for better risk stratification in patients who undergo these interventions. Table 4 summarized all findings.

**Table 4:** Predictive value of RV longitudinal strain in patients with valvular heart disease.

**References**	**Sample size**	**RV free-wall/global GLS cut-off**	**Follow-up period (months)**	**Main findings**
**Aortic stenosis**				
Kempny et al. (36)	123	–	3	RV free-wall longitudinal strain before and 1 year after TAVR or SAVR showed that RV longitudinal strain did not significantly improve after TAVR, but it significantly deteriorated in SAVR group.
Balderas-Muñoz et al. (37)	75	−15%	1	Patients after SAVR revealed that RV free-wall longitudinal strain >-15% had high sensitivity and specificity for development of low output cardiac syndrome in the first 30 days after surgery.
Posada-Martinez et al. (38)	75	−17.3%	24	RV free-wall longitudinal strain was independent predictor of low output cardiac syndrome, but not of in-hospital mortality, hospital stay, or vasoplegic syndrome.
Vizzardi et al. (39)	56	−17%	120	RV global longitudinal strain and RV-arterial coupling provided better risk stratification than other RV echocardiographic parameters in TAVR patients during long-term follow-up.
**Mitral regurgitation**				
Orde et al. (40)	158	–	36	The patients who underwent robotic-assisted mitral valve repair also showed greater recovery in RV longitudinal strain at 1-year follow-up comparing with pre-surgery values.
Chang et al. (41)	108	–	31	Only resolution of RV longitudinal strain at 1 month predicted the subsequent myocardial recovery. TAPSE, FAC, and s′ did not have any role in this prediction.
Elgharably et al. (42)	568	–	76	RVEF decreased, while LVEF increased during after concomitant surgery for ischemic mitral and tricuspid regurgitation. RV longitudinal strain showed continuous deterioration during follow-up period.
**Tricuspid regurgitation**				
Bannehr et al. (43)	1,089	−18%	24	Reduced RV free-wall longitudinal strain, TAPSE and FAC were independent predictors for all-cause mortality. The sensitivity and specificity to predict mortality gradually increased from FAC, across TAPSE, to RV longitudinal strain.
Prihadi et al. (22)	896	−23%	34	RV free-wall longitudinal strain was independently associated with all-cause mortality and incremental to FAC and TAPSE.
Ancona et al. (13)	250	–	24	The authors found that RV free-wall strain >-17% at baseline predicted RV heart failure, whereas patients with RV free-wall strain <-14% at follow-up had significantly better survival.
Romano et al. (44)	544	−16%	72	CMR-derived RV longitudinal strain was independent predictor of mortality after adjustment for clinical and imaging risk factors, including RV size and ejection fraction.

*CMR, cardiac magnetic resonance; CV, cardiovascular; FAC, fractional area change; GLS, global longitudinal strain; RV, right ventricle; s′, systolic velocity of the lateral segment of tricuspid annulus; SAVR, surgical aortic valve replacement; TAVR, transcatheter aortic valve replacement; TAPSE, tricuspid annular plane systolic excursion*.

### Aortic Stenosis

Study that investigated RV free-wall longitudinal strain before and 1 year after transcatheter or surgical aortic valve replacement (TAVR or SAVR) showed that RV longitudinal strain did not significantly improve after TAVR, but it significantly deteriorated in SAVR group, even though TAVR group had worse baseline clinical characteristics (more patients in NYHA class III and IV and significantly higher EuroSCORE) (36). However, it must be noted that RV strain improved in TAVR group for 2.4% in absolute values, which was still not enough to reach statistical significance in a relative small sample (*n* = 101). Interestingly, TAPSE and FAC also significantly reduced after SAVR and remained the same in TAVR group (36). This difference potentially could be due to pericardiotomy during SAVR. Another investigation that included patients after SAVR revealed that RV free-wall longitudinal strain >-15% had high sensitivity and specificity for development of low output cardiac syndrome in the first 30 days after surgery, which may qualify RV strain for risk stratification undergoing SAVR (37). The same group of authors recently provided results form long follow-up and showed that RV free-wall longitudinal strain was independent predictor of low output cardiac syndrome, but not of in-hospital mortality, hospital stay, or vasoplegic syndrome (38).

Dahou et al. (63) investigated patients with low-flow low-gradient aortic stenosis and found that 2-year survival in patients with RV free-wall longitudinal strain >-13% was significantly lower than in those with RV free-wall longitudinal strain <-13%. RV free-wall longitudinal strain >-13% was an independent predictor of mortality in these patients, regardless age, aortic stenosis severity, myocardial infarction and LV longitudinal strain (63). Stress RV longitudinal strain, measured during low dose dobutamine stress, has incremental prognostic value over LV longitudinal strain, LVEF and RV strain at rest (63) (Table 4).

Research that included HF patients due to severe aortic stenosis undergoing TAVR reported that RV global longitudinal strain and RV-arterial coupling (TAPSE/sPAP and RV longitudinal strain/sPAP) provided better risk stratification than other RV echocardiographic parameters during long-term follow up of 10 years (39). TAPSE, FAC, s′, sPAP, tricuspid regurgitation and tricuspid annulus diameter were not proven to be the independent predictors of 10-year mortality after TAVR (39).

Recent large study that involved 334 patients who underwent TAVR reported no significant change in RV free-wall longitudinal strain at 1-year follow-up, in both groups with baseline impaired and normal RV systolic function (12). However, RV longitudinal strain was associated with 1-year all-cause mortality. The authors found a 6% higher risk for 1-year mortality for each unit increase in RV longitudinal strain. Other parameters of RV systolic function (TAPSE, FAC, s′), as well as LVEF, existence of tricuspid regurgitation and sPAP were not independent predictors of 1-year mortality in these patients (12). These findings support the routine assessment of RV longitudinal strain in patients undergoing SAVR or TAVR in order to improve the risk stratification (Table 4).

### Mitral Regurgitation

RV systolic function is a very important in patients undergoing mitral valve surgery and some studies showed its strong predictive value for long-term mortality in a large population of patients who underwent cardiac surgery (valve, valve + CABG, CABG) (64). RV systolic function, assessed by TAPSE, FAC, and s′, remained depressed shortly after mitral valve surgery (65).

Study that involved patients who underwent mitral valve surgery reported significant deterioration of RV free-wall longitudinal strain in these patients (40). The authors found smaller reductions in patients who underwent robotic-assisted mitral valve repair than in patients who underwent standard mitral valve repair. The patients who underwent robotic-assisted mitral valve repair also showed greater recovery in RV longitudinal strain at 1-year follow-up comparing with pre-surgery values (40). Similar investigation that involved patients who underwent surgical mitral valve repair showed that only resolution of RV longitudinal strain at 1 month predicted the subsequent myocardial recovery (41). TAPSE, FAC, and s′ did not have any role in this prediction. Patients with preserved RV systolic function had a lower risk of hospitalization due to HF compared to those with reduced RV function (41) (Table 4).

Large research that involved 568 patients with severe ischemic mitral regurgitation who underwent surgical valve repair and 131 patients with concomitant tricuspid valve repair demonstrated that the RV continued to dilate while the LV reduced during follow-up after surgery (42). RVEF decreased, while LVEF increased during 6.3-year follow-up. RV longitudinal strain showed continuous deterioration during follow-up period. The largest decline in RV longitudinal strain occurred in the first month after surgery, while it remained constant after first year of follow-up (42). RV longitudinal strain, FAC and TAPSE were significantly lower in patients who underwent tricuspid valve repair than in those who did not undergo tricuspid valve repair. RV strain and FAC did not significantly recover during follow-up, while TAPSE significantly improved in both groups—with and without tricuspid valve repair. These findings showed that surgical treatment of functional mitral and tricuspid regurgitation along with revascularization failed to induce improve RV function and reverse remodeling (Table 4).

Functional mitral regurgitation has been currently treated with interventional “edge-to-edge” technique and study that investigated patients with moderate and severe mitral regurgitation, who underwent MitraClip procedure, demonstrated no acute improvement (within 7 days) in CMR-derived RVEF, RV end-diastolic volume and tricuspid regurgitation (66).

Findings from aforementioned studies warrant further examinations to identify optimal timing and approach of intervention for functional mitral and tricuspid regurgitation.

### Tricuspid Regurgitation

Isolated or concomitant functional tricuspid regurgitation has been extensively investigated in last decade. It has been shown that tricuspid regurgitation significantly deteriorated the outcome in patients with mitral regurgitation. However, surgical intervention seems not to improve RV function in these patients (42). Interventional “edge-to-edge” repair significantly increased interest for predictors that may improve outcome after this intervention. Hirasawa et al. (67) reported that reduction of RV free-wall longitudinal strain was proportional to the level of tricuspid regurgitation. Figure 1 shows difference in RV global longitudinal strain between control subjects and patients with severe functional tricuspid regurgitation.

Large study that included 1,089 patients with tricuspid regurgitation showed that reduced RV free-wall longitudinal strain (>-18%), TAPSE (<18.5 mm) and FAC (<35%) were independent predictors for 2-year all-cause mortality (66). The sensitivity and specificity to predict 2-year mortality gradually increased from FAC, across TAPSE, to RV longitudinal strain (43). Similar study that included 896 patients with significant tricuspid regurgitation defined reduced RV function as TAPSE <17 mm, FAC <35%, and RV free-wall longitudinal strain >-23% (22). Mortality was significantly higher in patients with decreased FAC, decreased TAPSE, and impaired RV free wall longitudinal strain. Multivariate analysis showed that RV free-wall longitudinal strain was independently associated with 2.8-year all-cause mortality and incremental to FAC and TAPSE (22). Reduced TAPSE and FAC also represented independent predictors for mortality after adjustment for age, LVEF, RV systolic pressure, significant left-sided valvular disease (22). However, after adjustment for concomitant diseases (diabetes mellitus, chronic kidney disease, coronary artery disease) as well as New York Heart Association class III/IV this independent predictive value vanished for FAC and TAPSE, but not for RV free-wall strain (22) (Table 4).

Investigation that enrolled 250 consecutive patients with severe tricuspid regurgitation investigated RV global and free-wall longitudinal strain and reclassified ~42–56% of patients with normal RV systolic function according to conventional parameters in patients with impaired RV systolic function (13). The authors found that RV free-wall strain >-17% at baseline predicted RV heart failure, whereas patients with RV free-wall strain <-14% at follow-up had significantly better survival (13) (Table 4).

Recent study included 544 patients with severe functional tricuspid regurgitation with a median follow-up of 6 years (43). Patients with CMR-derived RV free-wall longitudinal strain >-16% had significantly lower survival compared with those with lower RV free-wall longitudinal strain (44). CMR-derived RV longitudinal strain was independent predictor of mortality after adjustment for clinical and imaging risk factors, including RV size and ejection fraction (Table 4).

Current studies support the evaluation of RV longitudinal strain due to its high predictive value in patients with tricuspid regurgitation. Data about the effect of interventional “edge-to-edge” tricuspid valve repair are expected and they will provide some new guidelines if RV longitudinal strain should be involved in routine echocardiographic examination and it seems that body of evidence that supports this evaluation is constantly enlarging.

## Conclusion

RV longitudinal strain is a significant parameter of RV dysfunction that showed better sensitivity than conventional echocardiographic parameters. Latest investigation demonstrated its role in prediction of adverse outcome in wide range of CV conditions, but the most encouraging data came from studies that were focused on pulmonary hypertension, HFrEF and tricuspid regurgitation. Data about the predictive importance of RV longitudinal strain in HFpEF patients and those with aortic stenosis and mitral regurgitation are still scarce and warrant further investigation. Adding RV longitudinal strain to existing scores might significantly help in the risk stratification, which could induce reclassification of some patients to higher risk group. Existing knowledge about the predictive value of RV longitudinal strain supports its routine echocardiographic evaluation in patients with CV diseases.

## Author Contributions

MT wrote the article. NN, LS, JK, and CR reviewed the literature. JK made figures. DB, BG, and DS helped in preparing the text of the manuscript. EB, CC, and WR reviewed the article with important scientific input. All authors contributed to the article and approved the submitted version.

## Conflict of Interest

The authors declare that the research was conducted in the absence of any commercial or financial relationships that could be construed as a potential conflict of interest.
